# From manual clinical criteria to machine learning algorithms: Comparing outcome endpoints derived from diverse electronic health record data modalities

**DOI:** 10.1371/journal.pdig.0000755

**Published:** 2025-05-14

**Authors:** Shreya Chappidi, Mason J. Belue, Stephanie A. Harmon, Sarisha Jagasia, Ying Zhuge, Erdal Tasci, Baris Turkbey, Jatinder Singh, Kevin Camphausen, Andra V. Krauze

**Affiliations:** 1 Radiation Oncology Branch, Center for Cancer Research, National Cancer Institute, National Institutes of Health, Bethesda, Maryland, United States of America; 2 Department of Computer Science and Technology, University of Cambridge, Cambridge, United Kingdom; 3 Artificial Intelligence Resource, Center for Cancer Research, National Cancer Institute, National Institutes of Health, Bethesda, Maryland, United States of America; 4 Research Center Trustworthy Data Science and Security, University Alliance Ruhr, Duisburg-Essen, Germany; King’s College London, UNITED KINGDOM OF GREAT BRITAIN AND NORTHERN IRELAND

## Abstract

**Background:**

Progression free survival (PFS) is a critical clinical outcome endpoint during cancer management and treatment evaluation. Yet, PFS is often missing from publicly available datasets due to the current subjective, expert, and time-intensive nature of generating PFS metrics. Given emerging research in multi-modal machine learning (ML), we explored the benefits and challenges associated with mining different electronic health record (EHR) data modalities and automating extraction of PFS metrics via ML algorithms.

**Methods:**

We analyzed EHR data from 92 pathology-proven GBM patients, obtaining 233 corticosteroid prescriptions, 2080 radiology reports, and 743 brain MRI scans. Three methods were developed to derive clinical PFS: 1) frequency analysis of corticosteroid prescriptions, 2) natural language processing (NLP) of reports, and 3) computer vision (CV) volumetric analysis of imaging. Outputs from these methods were compared to manually annotated clinical guideline PFS metrics.

**Results:**

Employing data-driven methods, standalone progression rates were 63% (prescription), 78% (NLP), and 54% (CV), compared to the 99% progression rate from manually applied clinical guidelines using integrated data sources. The prescription method identified progression an average of 5.2 months later than the clinical standard, while the CV and NLP algorithms identified progression earlier by 2.6 and 6.9 months, respectively. While lesion growth is a clinical guideline progression indicator, only half of patients exhibited increasing contrast-enhancing tumor volumes during scan-based CV analysis.

**Conclusion:**

Our results indicate that data-driven algorithms can extract tumor progression outcomes from existing EHR data. However, ML methods are subject to varying availability bias, supporting contextual information, and pre-processing resource burdens that influence the extracted PFS endpoint distributions. Our scan-based CV results also suggest that the automation of clinical criteria may not align with human intuition. Our findings indicate a need for improved data source integration, validation, and revisiting of clinical criteria in parallel to multi-modal ML algorithm development.

## Author summary

Progression free survival is an important outcome in cancer research used to evaluate new treatments. However, this data is often not publicly available as it requires labor-intensive, subjective judgement from clinicians. Different data modalities, such as text reports and imaging, stored in the electronic health record could be used to automate the extraction of progression events from a patient’s medical record. This paper explores three automated and/or machine learning (ML) methods to extract progression from integrated electronic health data, including 1) analysis of patient prescription frequencies, 2) natural language processing algorithms applied to radiology reports, and 3) computer vision tumor segmentation algorithms applied to brain MRI scans. These automated results were compared to the current manual clinical standard method of determining progression. Our study found that various ML algorithms can automate the extraction of progression outcomes from diverse patient data. Yet, manual evaluation identified progression at a higher rate compared to data-driven algorithms. Our results indicated that “ground truth” labels obtained for training ML algorithms are influenced by both the data source and method used to obtain them. Future research should consider that varying data sources, availability, and reliability can create methodological bias during ML projects.

## Introduction

Glioblastoma multiforme (GBM), a form of high-grade glioma, is amongst the most aggressive brain tumors with a median survival 14 months [1]. Yet, brain tumor outcomes have seen limited improvement despite ongoing imaging, radiation therapy, and systemic management advancements. The ability to identify biomarkers associated with progression and treatment response is limited by data that often only includes survival as outcome endpoints.

Overall survival (OS) is commonly employed in patient datasets given its simpler calculation from date of diagnosis to date of death. However, OS is an imperfect outcome endpoint as it reflects the summation of multiple interventions beyond standard of care (SOC) upfront chemoirradiation (CRT), such as potential re-resection and use of study agents upon recurrence. Conversely, progression free survival (PFS), defined as the time between diagnosis to disease progression, is derived from a complex set of data sources using a subjective, labor-intensive process that surveys a patient’s medical record [2]. PFS data is instrumental for guiding disease management and biomarker research as it can indicate treatment response or failure, allowing for rapid intervention to treat lower tumor burdens or initiation of novel treatment options [3].

### Clinical standards for generating PFS data

Current neuro-oncology practice standards involve using Response Assessment in Neuro-Oncology (RANO) criteria to determine progression for glioma patients [2]. These criteria allow for a combination of clinical and imaging features. Progression is defined by Wen *et al*. [2] as including any of the following factors:

1. ≥25% increase in T1 gadolinium enhancing disease,
2. increasing T2/FLAIR volume,
3. any new lesions, and/or
4. deteriorating clinical status.

Determining true progression in glioma is difficult due to the temporary clinical and radiographic deterioration that patients may experience following completion of CRT. This deterioration is termed *pseudoprogression* if these symptoms result from acute effects of management and reduce over time [4–6]. While previous RECIST progression criteria did not account for deteriorating clinical factors, the MacDonald criteria update eventually incorporated clinical status and corticosteroid administration [7] and successive RANO iterations added caveats for pseudoprogression. Despite these changes, there are still limitations towards obtaining consensus on tumor progression. For example, progression of disease is based on a ≥25% or greater increase in the product of perpendicular diameters on contrast enhanced imaging, which can be subjective and represent pseudoprogression without changes outside the radiotherapy (RT) field. Moreover, it should be noted that the extent and location of the RT dose cloud is not readily available for visualization to radiologists or even some neuro-oncology teams. Thus, tumor size or lesion counts are often not explicitly captured or recorded in a patient’s electronic health record (EHR). While ongoing revisions to RANO currently include adapting to the use of immunotherapeutics and molecular disease classification [8], there are still limitations in the quantification of tumors identified by imaging.

Given the aforementioned limitations, non-clinical-trial glioma data sets do not have a straightforward progression date for patient unless retrospectively assigned in small cohorts. Most publicly available brain tumor data sets do not include PFS data, including The Cancer Genome Atlas (TCGA) [9], The Cancer Imaging Atlas (TCIA) [10], Georgetown Database of Cancer (G-DOC) [11], and the Chinese Glioma Genome Atlas (CGGA) [12].

### Data integration and multi-modal machine learning (ML)

Clinical application of RANO criteria involves review of multiple EHR data sources and modalities by skilled clinicians. This process reflects human attempts to integrate and extract insights from multiple modalities of medical data, including scans, radiology reports, progress notes, and other clinical context over time and potentially institutions. ML research has begun to focus on multi-modal algorithms with the goal of more closely aligning with clinical practice, where a totality of information is processed during diagnosis and treatment [13, 14]. Some studies have often demonstrated that multi-modal algorithms demonstrate superior performance over unimodal algorithms trained on a singular stream of data [15, 16]. However, it is not yet clear how various data modalities may influence the predictions of a multi-modal algorithm, either due to the information encoded within the data or biases surrounding the data collection process. As a result, data integration for multi-modal ML analysis has remained underexplored, particularly in the areas of endpoint extraction and brain cancer.

Recent attempts to compute PFS metrics from data using artificial intelligence (AI) have successfully used clinical features [17] and radiomic features extracted from brain MRI scans, including texture and morphological features [18] and quantified tumor volumes [19]. While Kwiatkowska-Miernik *et al*. [18] identify that four out of six of their models demonstrate appropriate predictive performance via mean absolute percentage error, they do not detail specific criteria applied to obtain ground truth progression (“determined based on follow-up MRI exams evaluated by an experienced radiologist”) and only evaluate a cohort of 51 patients meeting their inclusion criteria. Meanwhile, Kickingereder *et al*. obtain 87% agreement between automated neural network versus radiologist drawn volumes; however, this volumetric approach demonstrated lower agreement (between 73% to 51% depending on the test set) with manually applied RANO criteria, indicating a need to explore other volumetric approaches or definitions [19].

At the same time, others suggest that these ML outcome prediction studies may lack complete inclusion of histologic, pathologic, and molecular data sources that mirror clinical practice [20]. Some retrospective analyses on clinical GBM data sets have integrated imaging data sources including histopathology imaging [21] and genetic alterations [22]. Yet, these studies generally do not study overall or progression free survival as an outcome endpoint. Clinical practice guidelines currently do not stipulate PFS capture by means other than manually applied RANO criteria. Thus, there is a need for approaches to increase PFS availability and further mine for linkages between progression and imaging, -omic, and other clinical features.

### Data capture in the electronic health record during cancer treatment and management

**Fig 1:**
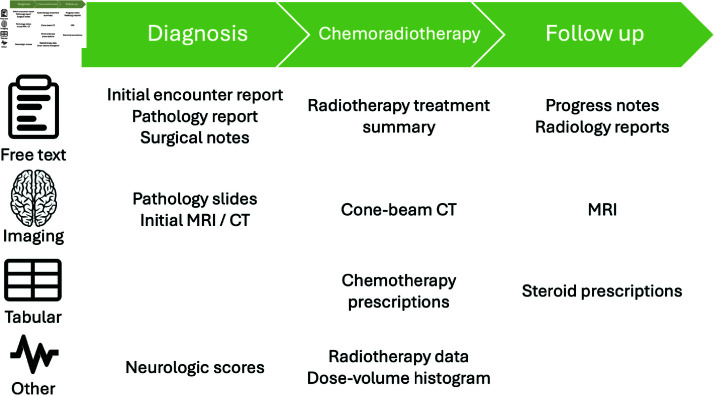
Sample cancer patient treatment timeline with data generated and captured within the EHR.

Several clinical data elements are collected and stored over the natural history course of a patient’s cancer diagnosis (Fig 1). The following subsections discuss cancer standard of care and corresponding diverse data sources that could be used to obtain progression free survival.

#### Clinical standard of care.

For glioblastoma multiforme (GBM), treatment standard of care involves maximal surgical resection followed by radiotherapy (RT) with administration of concurrent and maintenance temozolomide (altogether termed chemoirraditation (CRT)). Following completion of CRT, patients are followed clinically with contrast-enhanced MRI completed 2-8 weeks post CRT, then repeated every 2-4 months for 3 years, and then every 3-6 months indefinitely per national and international guidelines [23].

#### Tabular prescription data.

GBM patients often experience devastating neurological symptoms and are usually prescribed corticosteroids to manage these acute effects. Corticosteroids act by decreasing inflammation in the brain and may be administered prior to surgical intervention, post-surgical intervention (most common), during CRT, following completion of CRT to manage acute effects, or upon tumor progression. Oral dexamethasone is the most commonly prescribed, while intravenous loading may be selected when a more rapid effect or loading dose is indicated. A “tapering schedule” for gradual discontinuation of dexamethasone is employed to mitigate potential adrenal insufficiency and worsening of neurological symptoms. Corticosteroid prescriptions are captured in the EHR and their use can theoretically be correlated with radiographic report findings and clinical records. However, there is widespread heterogeneity in prescription patterns and tapering schedules, as well as subjectivity involved in the initiation of steroids. Thus, steroid usage is often difficult to implement and retrospectively interpret.

#### Free text documents.

Hundreds of documents can be generated over the course of a patient’s cancer diagnosis and treatment (Fig 1). EHR systems are often dated and lack infrastructure to share information with other systems which limits bulk and longitudinal analysis. Free text documents held within the EHR are often reviewed manually by a clinician prior to a patient’s visit or update in care. However, this process can be repetitive, time-consuming, and prone to error as details may be omitted or redundant between documents. As a result, natural language processing of clinical documents has been an increasingly popular method to improve efficiency of medical record analysis.

#### Imaging.

Numerous medical images from various imaging modalities are collected over the course of a patient’s diagnosis, treatment, and care management, including magnetic resonance imaging (MRI), computer tomography (CT), and cone-beam CT scans (Fig 1). However, cone-beam CT scans are not typically available outside of the radiation oncology department where they are used for treatment verification. Moreover, while RANO criteria indicate a quantitative metric to observe 25% volume increases in enhancing lesions, in practice, it is not common practice to quantify lesions or other enhancing regions identified on MRI scans, especially in community or non-neuro-oncology specialized settings. Moreover, when measurements are obtained, the rate of agreement between radiologists is generally ≤50% which limits the utility of these metrics during analysis [24]. In addition, radiologists are generally not privy to radiation treatment dose cloud data, such as the 80% isodose line which can indicate recurrent disease outside of the high dose field, making it more difficult to distinguish *pseudoprogression* from progression given any increased enhancement [6].

#### Human influences on EHR data.

Automated methods to derive data outcome labels may not necessarily “objective” as EHR data sourecs are subject to inclusion bias, representativeness issues, and other types of biases [25, 26]. The increased accessibility of radiology reports compared to their source imaging files could make a free-text, natural language processing (NLP)-based method for obtaining progression metrics more desirable due to increased data point availability within a given patient timeline. However, radiology reports usually reflect a single author’s judgment based on the medical conventions of the time [27] and studies document differences in interrater reliability during imaging analysis [28]. Sole reliance on radiology reports can create positive bias in a reconstruction of a patient’s medical history from EHR data, as we only have access to what was explicitly measured and included in the report [25, 26, 29]. Human-generated textual data can mirror issues with the “file drawer problem” in scientific publishing where information deemed as non-notable cannot be accessed by other potentially interested parties [30]. This reflects a common tension in medical machine learning (ML) where data annotation requirements for ML include information about the presence and absence of every possible diagnostic option, as opposed to clinical practice, where clinicians usually only document notable features that require further attention or potential follow-up [27]. Other datapoints, such as MRI scan frequency and acquired scan parameters, are also constrained by provider-based practices at the time including follow-up frequency and machine availability.

### Automated approaches to derive PFS

Over the last decade, the medical field has seen an explosion in accessible and queryable EHR data, though there are still large gaps in retroactively transferring older patient data and integrating various sources. Barriers to digitization of medical data also persist, including fear of documentation due to stigma related to diagnosis and treatment of certain diseases such as HIV [31]. The subjective and labor-intensive process of generating annotations for supervised machine learning has also highlighted issues such as label bias and low inter-rater reliability [28, 32, 33]. These issues have led to increased interest in ML label generation methods, though current annotation algorithms carry their own set of issues, including narrower labeling abilities and technical onboarding challenges [34, 35]. Given critical challenges in generating clinically-relevant labels/annotations for supervised machine learning, we discuss and survey the current literature on automated approaches to generate outcome endpoints using EHR patient data.

#### Natural language processing (NLP).

Natural language processing (NLP) algorithms attempt to understand human-generated text by computationally encoding and representing text [36]. A large portion of current NLP research is centered on text generation [37] and knowledge checking [38, 39] due to current advances in large language models (LLM); however, there is growing literature focused on extracting structured details from unstructured free text in applications including multiple sclerosis traits [40], chronic disease [41], activities of daily living [42], social determinants of health [43, 44], and other clinical traits [45–48]. Rule-based NLP approaches capitalize on domain knowledge by matching to human-specified keywords or patterns in text [36]. In contrast, other deep learning approaches tend to employ more complex algorithm architectures to predict or classify text based on larger training data sets and concept-level annotations [36].

In the context of cancer care and management, NLP has been used to extract pathological information for prostate cancer [49], BI-RADS assessments from radiology reports in breast cancer [50], initial treatment types [51], breast cancer phenotypes [52], and other quantitative clinical information [53]. A scoping review of 123 publications by Wang *et al*. [54] found that most cancer-related NLP algorithms were built with the aims of general information extraction and cohort identification, with only 3 studies attempting to visualize disease history and the authors explicitly identifying outcome analysis as a current gap in NLP-assisted mining of EHR text data.

For outcome identification, NLP algorithms have been deployed to identify recurrence in breast cancer [55, 56], response events and progression events in lung cancer [57], progression using structured and embedded free text in glaucoma [58], and progression across cancer types using EHR-derived Framingham risk scores [59]. Sangariyavanich *et al*. [36] conduct a systematic review of 267 models across 17 studies using NLP to identify recurrent cancer, with a majority relying on statistical text representation. The authors find slightly superior performance between studies using deep learning NLP compared to rule-based algorithms, but acknowledge a lack of comparative literature in developing and deploying algorithms to detect recurrence or progression.

Most papers reviewed by [36] evaluate algorithm performance through calculated area under the receiver operating curve (AUROC), F1, precision, and/or recall scores, requiring manually curated ground truth data sets to identify report-level labels of either recurrence or stable disease. The review reports median F1 scores of 0.71, 0.43, and 0.76, for the rule-based, ML, and deep learning approaches evaluated, respectively [36]. However, given current challenges in medical data sharing, there are little to no publicly available datasets with report-level progression annotations for cross-validation. At the time of this publication, there are also few studies investigating suitable proxies for progression via free text or NLP methods. Thus, the current state of NLP-supported structured endpoint extraction relies on hand-crafted, report-level ground truth, which is time-intensive to curate and not often shared for further validation.

Outside of predictive performance, NLP algorithms may also be evaluated in other dimensions including algorithmic complexity, privacy and security, interpretability, and veracity. While deep learning algorithms may often achieve comparable [60, 61] or superior accuracy [36, 62] to rule-based approaches, they are often subject to differences in required resources for training and deployment, training data set sizes, developer and clinician user familiarity, output verification processes, privacy and security concerns, and methods to achieve interpretability [60, 61]. Berge *et al*. [61] emphasize the specific need for local approaches in the medical domain, which motivates the use of rule-based approaches or transfer learning in the context of larger foundation models for deep learning approaches. Bhattarai *et al*. [62] also note that outputs from local rule-based models such as spaCy are also deterministic (compared to emerging LLM approaches using models including GPT-4 which provide non-deterministic outputs without current widely accepted gold standard methods for verification).

#### Computer vision (CV).

Computer vision is a field of computer science dedicated to extracting information from visual or image data. There is extensive literature dedicated to machine learning pre-processing and processing of MRI scans [63]. Many of these applications involve signal processing, segmentation, auto-contouring, and other disease detection algorithms. However, brain scans require additional processing for anonymization/de-identification purposes, which represents a barrier to public data sharing [64]. Thus, there are also few studies aimed at quantifying and tracking progression in brain tumors directly via imaging.

Direct volumetric imaging analysis may appear to be a more “objective” method to determine tumor progression. However, medical image processing is a far more resource- and expertise-intensive task that can conflict with changing and evolving technologies in image processing and data storage over time. Even with a sufficiently large imaging data set, pre-processing is a labor- and time-intensive task requiring several registration, skull-stripping, contouring, and de-anonymization steps to allow for comparisons within and between patients. While extra steps can be taken to integrate and share scans between institutions, including federated learning initiatives [65], current computer vision (CV) research indicates reduced transferability and generalizability of ML-based decision-assisting algorithms when patient scans are obtained from different imaging machines and facilities [66–68].

Some studies have explored the use of data-driven algorithms to detect tumor features, including primary gross tumor volume (GTV) contouring in patients with nasopharyngeal carcinoma [69] and peritumoral edema in recurrent GBM [70]. A review of literature linking radiomic features to other biomarkers [71] found three studies linking lesion or necrotic volume to genetic features, but none of the cited studies explored ML segmentation algorithms or linked data to outcomes [72–74]. Kidd *et al*. [75] used convolutional neural networks to extract volumes from malignant pleural mesothelioma patients and compare against modified RECIST (Response Evaluation Criteria in Solid Tumors) criteria, finding a significant difference in AI-derived volume changes between partial response and progression patients. These studies indicate the need for further exploration and validation of automated tumor segmentation volumes, particularly when linking to clinical features and outcomes.

### Contributions

Given current barriers in generating PFS data in the context of GBM, this paper aims to mine, integrate, and automate large-scale EHR data to arrive at PFS endpoints efficiently, and compare automated and/or machine learning PFS endpoints to manually-derived PFS metrics using clinical guidelines. This data integration framework can be replicated to add PFS outcomes in other large-scale data sets given acute clinical need and lack of data availability in other cancer disease sites and medical disciplines [19, 76, 77]. In this paper, we showcase:

1. the integration of clinical, imaging, and prescription medication data within a queryable framework;
2. the automated identification of a progression free survival date using corticosteroid administration, natural language processing (NLP)-analyzed radiology reports, and computer vision (CV)-derived MRI tumor volumes;
3. with comparison to manual chart review as the clinical gold standard for progression according to RANO.

## Materials and methods

This analysis set out to mine various EHR data modalities, automate the extraction of PFS metrics via ML algorithms, and evaluate the ability of these methods to extract relevant progression evidence from a given modality compared to the current clinical standard approach using manually RANO criteria.

### Patient cohort

**Fig 2:**
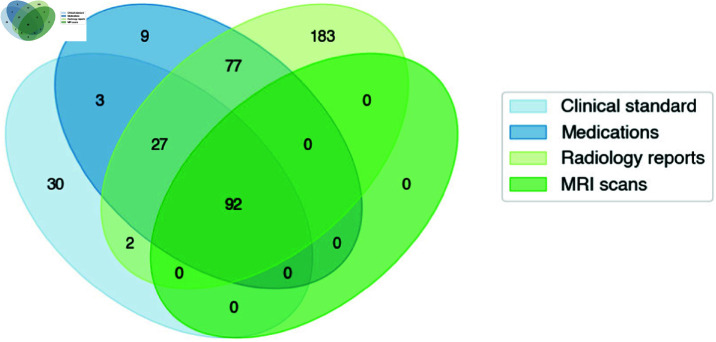
Overall patient cohort with overlapping data source availability.

The patient cohort initially included 423 brain malignancy patients who received treatment on protocol at the NIH. All patients were treated on NCI NIH IRB (IRB00011862) approved protocols. Given the significant radiographic differences between GBM and lower grade glioma patients, the current analysis focused on patients with GBM confirmed via histopathology to ensure homogeneity. Patients were excluded if a manual progression date could not be determined due to loss to follow-up or patient expiration without overt progression evidence (i.e. death occurring from non-glioma cause or less then 1 month following completion of CRT) (Fig 2). Patients were also excluded for lacking at least one queryable radiology report, one medication prescription, and two brain MRI scans (for comparative purposes) dated after their completion of chemoirradiation. Data was aggregated and queried through the NIH Integrated Data Analysis Platform (NIDAP). Available patient data included demographic and clinical attributes, MRI reports, progress notes, lab results, medication lists, and imaging scans.

### Clinical standard for assigning PFS

**Fig 3:**
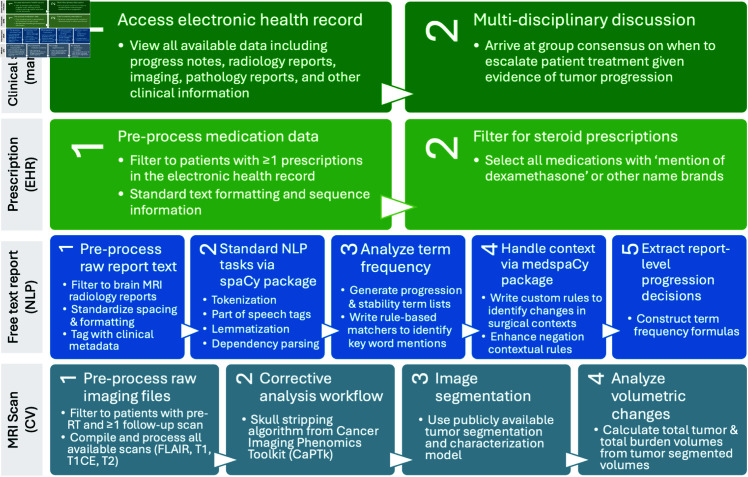
Paradigm for manual and automated methods to derive progression free survival.

A clinical standard progression date for each patient was assigned using via manual review of patient charts with RANO criteria progression (Fig 3). Progression in clinic was determined based on clinical and specifically neurological status, need for symptom management (e.g., use of steroids, recurrence of seizures requiring augmentation or initiation of seizure medication, etc.), and any alterations in patient status from previous functionality. These factors were concurrently considered by a clinical team with imaging alterations in tumor volumes treated with RT. Determination of progression was not the result of a single data modality or a single individual but rather the result of multidisciplinary discussion with consensus being reached after evaluation of all the features, which was then captured as progression in clinical progress notes. The nuances of this discussion are to some extent captured in clinical notes; however, data quantitatively documenting the number of individuals in the discussion (minimally ≥2 and typically >5) and their level of agreement are not captured. The consensus (agreement) of the group is based on real time application of RANO criteria and manually captured as consensus for progression or stability in this study. Other studies [27] have cited disconnects between the method and physical/virtual equipment used for ground truth labeling in clinical practice versus ML data annotation. Thus, to avoid this limitation, clinical standard RANO criteria were applied in the exact clinical context using the same equipment and software that providers used when treating patients.

### Corticosteroid administration analysis

All available prescriptions throughout a patient’s medical history were queried from the NIH Integrated Data Analysis Platform (NIDAP). Trends in prescription types, frequencies, doses, and sequences were analyzed. Prescriptions matching the generic key word ‘dexamethasone’ and associated brand names of any dosage and any administration route were selected for further analysis. Since GBM standard of care involves prescribing steroids immediately after surgery and CRT, analyses were limited to prescriptions dated 1 month after the end of CRT. Since steroids are prescribed on a tapering schedule, the first date of the largest dose prescription was followed continuously until the last date of the smallest prescription to determine the window of steroid tapering. During all subsequent analyses, this window was treated as a single course of steroids post-CRT.

The first date of the post-CRT steroids course was compared to the manually obtained clinical standard progression date. The number of prescriptions and months after treatment completion were compared to year of treatment to identify any department-level changes in prescription practices over time.

### Natural language processing of radiology reports

All available medical documents throughout the patient’s medical history were pulled from the integrated data framework. Free text document analyses were limited to brain MRI radiology reports.

Documents dated prior to the end date of a patient’s radiation therapy course were dropped to maintain consistency between variable-length patient histories. Document text was pre-processed to standardize paragraph formatting and spacing. The open-source https://spacy.io/Python spaCy package was used to perform standard natural language processing tasks including part of speech tagging, lemmatization, and dependency parsing (Fig 3). The add-on https://github.com/medspacy/medspacymedspaCy package was used for further handling of medical context and document section parsing [78].

Overall trends in word frequencies were analyzed within and across reports. We annotated the clinical standard RANO criteria for verbs and adjectives related to both progression and stability, as described in [79]. A trained clinician also viewed the descending term frequency list obtained from an aggregate of reports (S3a Fig) and sorted terms potentially relevant to determining progression criteria into either progression or stability categories, similar to the method employed by [80]. Using clinical standard RANO criteria and these observed frequency trends, we created a list of words hypothesized to indicate either progression or stability (listed in S1 Table).

Rule-based matchers with these term lists were created to search and tag any lemmatized instances of progression- and stability-related tokens within each document.

The medspaCy extension package was used to identify and handle contextual modifiers of these key terms, including negations and familial, historical, and hypothetical mentions. A custom ‘surgical’ contextual pipeline was constructed to match any tokens modified by surgical or postoperative terms to separate out tumor changes related to post-surgical effects of treatment. The ‘negation’ contextual pipeline was also expanded to include other terms commonly indicating no change in clinical practice given the high likelihood of radiology reports to indicate stability as a lack of positive findings (S1 Table).

Each patient document was processed via the custom spaCy and medspaCy NLP pipeline implementation, and progression- and stability-related terms were extracted and categorized per document. Progression terms modified by negated or historical contextual terms in the document were re-categorized as ‘stability’ terms. Progression terms modified by surgical context were dropped from the progression category term list due to their high likelihood of indicating *psuedoprogression* as compared to actual progression.

The frequency of progression-related words for a given document was compared to the frequency of stability-related words to determine the overall document status. A higher frequency of progression-related words indicated overall progression within the document. If the number of progression-related terms equaled the number of stability-related terms, then surgical-context modified terms were included in the analysis to provide additional context. Various weightings and thresholds for obtaining a report-level determination from each term categories were tested. We also tested various approaches to using RANO criteria as a proxy for report level ground truth (e.g., selecting all reports within a time window of clinician ground truth); however, given the goal of independently testing results derived from various data modalities, we wanted to avoid using the results of one modality to optimize or constrain the predictions of another modality (e.g., using manual ground truth to optimize weights for the NLP-based methods). Thus, given a lack of report-level ground truth and publicly available reports for validation tests, a one-to-one weighting was ultimately selected in this study. This weighting was selected with the goal of testing a rule that could be straightforwardly communicated to clinicians and with acknowledgment that alternate approaches should be evaluated and optimized in future work.

Report-derived progression dates were obtained by selecting the date of the first report that indicated progression overall based on the term frequency formulas described above. These report-derived progression dates were compared to both manually-obtained clinical standard dates and to other data-derived progression methods.

### Computer vision analysis of MRI scans

All available brain MRI imaging throughout the patient’s medical history were pulled from the integrated framework. Only patients with at least two post-RT scans were included. The following 3T MRI sequences were acquired: T1-weighted pre-contrast, T1-weighted post-contrast, T2-weighted, and T2-weighted fluid-attenuated inversion recovery (FLAIR). The complete methods for deriving the brain MRI volumes are further detailed and published in [81]. The tumor segmentation pipeline classified four tissue types: 1) background, 2) contrast-enhancing tumor, 3) non-contrast-enhancing tumor, and 4) edema.

Given that current clinical standard RANO criteria involve observing a 25% increase in contrast-enhancing lesions to indicate progression [2], we chose to limit our analysis of relative volume changes to **contrast-enhancing tumor**. Volumetric changes were calculated by dividing a given scan volume over the volume from the initial reference or baseline brain MRI scan available post-surgery but pre-CRT intervention Eq 1. To ensure adequate capture of alteration in contrast enhancement for patients with both large and small tumor volumes while also avoiding false positives created by small segmentation errors, we elected to treat a ≥5% increase in volume as an indication of progression.

$$\text{relative volumetric change}=\frac{\text{post-CRT contrast enhancing tumor volume}}{\text{baseline contrast enhancing tumor volume}}$$ (1)

Imaging-derived progression dates were obtained by selecting the earliest date of scans with a ≥5% relative increase in contrast-enhancing tumor volume. These image-derived progression dates were compared to manually-obtained clinical standard dates and compared to other data-derived progression methods.

### Comparative analysis

The data-derived progression methods were aggregated by patient for overall comparison and analyzed for statistically significant differences in the overall distributions and individual differences between data-derived dates. Given that not every patient met the criteria for progression under each progression method, many of these comparisons reflected a smaller subset of the overall cohort.

**Fig 4:**
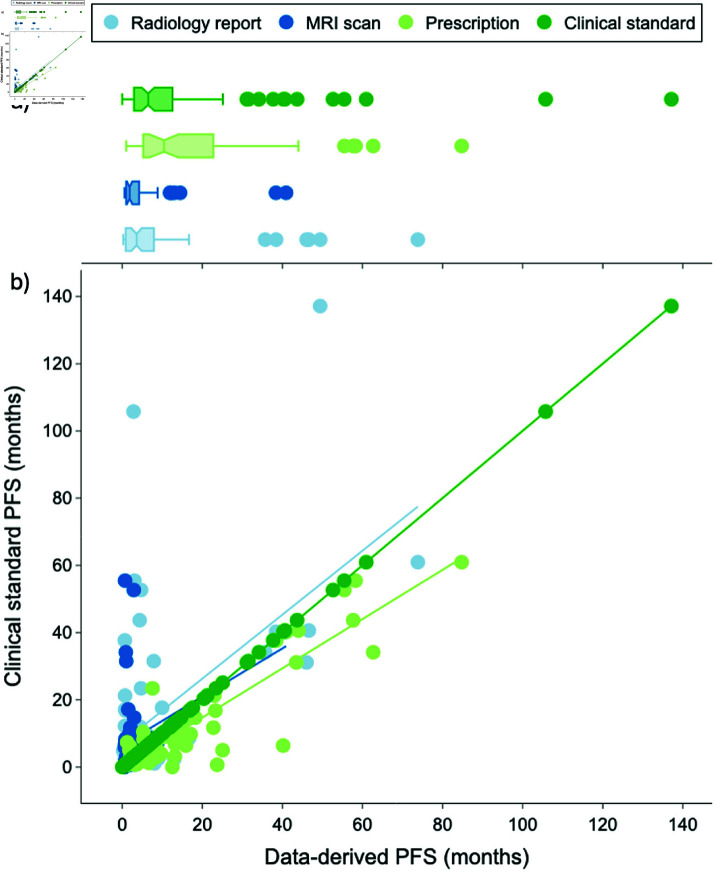
a) Boxplot and b) scatterplot distributions of manual and data-derived progression free survival dates. The dark green line represents the clinical standard PFS dates, with points falling above the dark green line indicating that the automated method derived an earlier PFS date compared to the clinical standard and points falling below indicating that the method derived a later PFS date. The light blue, dark blue, and light green trendlines reflect the Ordinary Least Squares linear regression for the radiology report, MRI scan, and prescription methods, respectively.

Non-parametric statistics were used to compare progression timeline dates given that the normality assumption for the progression date distributions was violated (i.e., very long-term survivors lead to a right-skewed distribution as seen in Fig 4a). The input data was the calculated PFS (in months) and the dependent variable was the method used to obtain the calculated PFS metric.

The Kruskal-Wallis Test was used to examine differences in datapoint progression timelines. The data met the test criteria as the observed PFS metrics (i.e., number of months) were continuous, the methods to obtain each PFS metric were not dependent on each other, and each method contained a sufficiently large number of positive observations.

The Wilcoxon signed-rank test with Bonferroni correction was used for pairwise comparisons between different datapoint timelines. The data met the test criteria as observations were 1) not normally distributed (Fig 4a), 2) dependent or naturally paired samples (i.e., each method calculated a PFS metric for the same given patient), and 3) independent from other pairs (i.e., metrics were calculated for each patient separately).

## Results

While the brain malignancy cohort receiving treatment at the National Institutes of Health (NIH) was around 423 patients, this analysis required integration of data from various sources. 331 patients were excluded for lacking either a confirmed GBM diagnosis or at least one instance of each EHR data modality queried in this paper. Ultimately, all four types of data were available for 92 patients receiving treatment between 2004-2023 at the NIH.

### Manual clinical standard

Following manual determination of patient progression using RANO criteria with MRI report and clinical exam review, 99% (n=91) of patients experienced tumor progression. These patients progressed an average 404 days or 13 months (stddev: 20.9 months) after the end of their last day of RT (Table 1).

**Table 1:** Descriptive statistics for a) manual and automated methods to derive PFS dates and b) relative differences between the manual PFS method and each automated PFS datapoint. PFS statistics are reported in months. Negative statistics indicate that the automated PFS date occurred prior to the manual PFS date.

		Clinical standard	Prescription	Radiology report	MRI scan
a)	**% progressed**	99% (n=91)	63% (n=58)	79% (n=73)	54% (n=50)
	**mean PFS**	13.3	17.9	8.0	6.2
	**std dev**	20.9	18.8	13.2	11.6
	**median PFS**	6.5	11.8	3.6	1.9
	**range PFS**	0-137.1	1-84.8	0.3-73.8	0.6-55.5
		**Prescription**	**Radiology report**	**MRI scan**	
	**mean PFS difference**	4.5	-6.9	-2.6	
b)	**std dev**	8.3	19.2	5.8	
	**median PFS difference**	2.5	-1.6	-0.03	
	**range PFS difference**	-6.1-33.8	-103.0-14.9	-33.5-2.1	

There was no association observed between the date of treatment received and length of clinical standard progression free survival timelines (*R*^2^ = 0.0,*F*(1,89) = 0.0004110,*p* = .865) (S1 Fig).

### Corticosteroid prescription analysis

**Fig 5:**
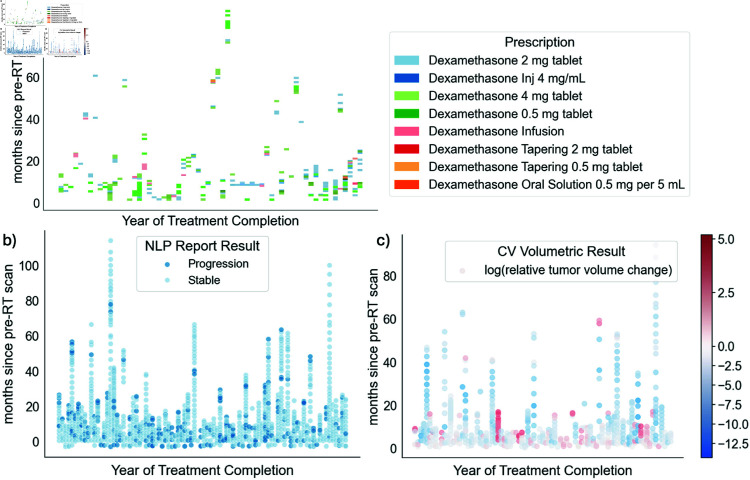
Patient timelines and progression results for available a) steroid prescriptions, b) radiology reports, and c) brain MRI scans. c) Red and blue points indicate scans with a relative increase and decrease, respectively, in contrast-enhancing tumor volumes compared to the baseline post-surgery, pre-RT scan.

23928 total prescription orders across the entire medical history of 92 patients were identified. 223 or 0.9% of these prescriptions across 58 patients were specifically for dexamethasone. Given the need for a tapering schedule for dexamethasone, patients often received multiple prescriptions of varying doses for a given “course” of steroids (Fig 5a). In comparison, 91 (99%) patients were identified as having progressed via the manual clinical standard method. The median date of these steroid prescriptions were 11.8 months after end of radiotherapy (stddev: 18.8 months). When compared to a given ground truth progression date for a patient, post-radiotherapy steroid prescriptions occurred an average of 4.5 months (range -6 to 34 months, median 2.5 months, stddev 8.3 months) after clinical standard progression dates.

### Natural language processing of radiology reports

1993 available radiology reports across 92 patients were identified. 1862 documents were dated on or after the patient’s diagnosis date, and 1677 documents were dated after a patient received CRT. Within reports dated after a patient’s diagnosis, the most common document types included “MRI BRAIN-Perfusion (IP)” (n=1245), “IP Perfusion” (n=90), “CT Cerebrum” (n=97), and “DX Chest - PA + Lat” (n=69). Only reports including brain MRI results after a patient’s diagnosis date were selected for further analysis.

A total of 1243 brain MRI radiology reports dated after treatment completion were available across 92 patients, with an average document length of 347 words. Frequency analysis indicated that the most common disease-relevant terms used in these reports included ‘enhancement’, ‘perfusion’, ‘enhancing’, ‘increased’, ‘tumor’, and ‘abnormal.’ In consultation with RANO criteria and frequency analysis of these documents, a list of words conceptually related to progression and stability were generated and used to write matcher rules for NLP-based text analysis. Using these lists, documents were analyzed for terms mentioned on either list (S3a Fig).

Terms indicating stability were mentioned 2641 times across 1243 documents, while words indicating progression were mentioned 1233 times (S3a Fig). 803 additional terms were related to progression but modified by surgical context. Documents often contained terms pertaining to multiple categories. 70% of documents contained at least one term related to ‘stable,’ and 35% of documents contained a term relating to both ‘stable’ and ‘progression.’ 11% of documents contained terms relating to ‘stable,’ ‘progression,’ and ‘surgical progression’ simultaneously.

After applying ‘progression’ and ‘stable’ category term-frequency formulas to each report, overall progression was identified in 222 reports (18% of post-RT reports) belonging to 73 patients (80%) (Fig 5b). The average date of these first progression reports were 8 months after end of radiotherapy (stddev: 13 months). When compared to a given ground truth progression date for a patient, radiology reports indicating progression occurred an average of 6.9 months (range -103 to 14.9 months, median -1.6 months, stddev 19 months) prior to clinical standard progression dates.

### Computer vision analysis of MRI scans

A total of 743 scans were available across all 92 patients following surgery. On average, edema was the largest identified volume, followed by non-contrast-enhancing tumor and contrast-enhancing tumor (S2 Table). Total tumor, defined as the sum between non-contrast-enhancing and contrast-enhancing tumor, displayed a right-tailed distribution of values with large variation in the fourth quartile (S4 Fig). Total burden, defined as the sum between total tumor and edema volumes, reflected a wider range of scan-level volumes.

To identify scans that indicated progression from a pre-RT baseline scan, the relative change in **contrast-enhancing tumor** was calculated between each baseline scan and subsequent follow-up scan. 134 (23%) scans across 52 (57%) patients indicated *any* increase in contrast-enhancing tumor volume from an initial baseline scan and 125 scans across 50 (54%) patients exhibited a ≥%5 increase. Given the wide range in patient brain volumes and volume changes, Fig 5c visualizes the logarithmic relative slope change in contrast-enhancing tumor for all available patient scans over time.

The average dates of the first progression-indicating scans were 6.2 months after end of radiotherapy (stddev: 11.6 months). When compared to a given ground truth progression date for a patient, scans with at least 5% increasing contrast-enhancing lesions occurred an average of 2.6 months (range -33 to 2.1 months, median -.03 months, stddev 5.8 months) prior to clinical standard progression dates.

### Comparative analysis

An average of 2.4 steroid prescriptions, 2.4 progression-indicating radiology reports, and 1.4 progression-indicating MRI brain scans were available per patient. The total months to first progression-indicating datapoint were compiled for each method in Table 1a and the relative time span compared to clinical standard were calculated for the three automated progression data methods in Table 1b.

There were significant differences observed between the four methods of determining progression for patients that progressed via all four methods $\left(\chi^{2}=39.7,p=1.2e\;-\;8\right)$. Post hoc pairwise comparisons showed significant differences between the clinical standard progression timelines and those obtained from scans with relative ≥5% increases in contrast-enhancing tumor volumes (W=133.0,p=5.7e−4), steroid prescriptions (W=234.5,p=2.555e−6), and radiology reports (W=672.5,p=.002) after Bonferroni correction. Progression dates derived from reports were significantly different from those derived from steroids (W=136.5,p=2.2e−06) but not scans (W=281.0,p=.418). All but one scan progression date occurred earlier than the respective steroid prescriptions for patients with both datapoints available (W=12.0,p=8.1e−09).

**Fig 6:**
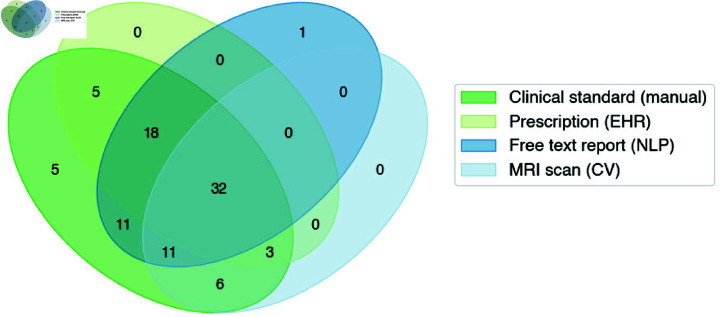
Progression-indicating datapoints for studied patient cohort.

Compared to the clinical standard method that identified progression in 99% (n = 91) of patients, the report NLP indicated the highest number of recurrent patients (n = 72), followed by steroid prescription analysis (n = 58), and lastly volumetric-based analysis of scans (n = 50) (Fig 6). The data modality that came closest to the clinical standard progression dates was steroids (avg 4.5 months later), followed by volumetric-based scan analysis (avg 2.6 months earlier), and then report-based NLP (avg 6.9 months earlier).

Fig 4 visualizes the boxplot and scatterplot distribution of progression dates for each method. The available data-derived progression dates were within 2 months of the clinical standard progression dates for 36% of report-derived dates, 66% of scan-derived dates, and 36% of steroid-derived dates.

## Discussion

We compare results between manual and different data-driven and/or machine learning methods to capture progression events using diverse data modalities within an integrated patient data framework.

### Clinical standard

RANO criteria is the current standard for determining progression for a given brain malignancy patient. Standard clinical application of RANO criteria involves review of multiple sources of medical data available to a specialized or skilled clinician. This process can be disrupted without complete compilation of scans, radiology reports, progress notes, and other clinical context over long periods and potentially across multiple medical institutions. Manual review of patient charts is also time consuming and labor-intensive. However, expert clinicians hold rich domain knowledge and can incorporate additional context and judgment available in clinic or during dual review of other chart elements.

Readers during the study noted difficulty in making definitive progression determinations during many patient cases. For example, sometimes progression would be indicated within a specific radiology report despite no changes made in a patient’s treatment protocol. This indicated that another clinician likely chose to follow-up and/or wait for further information before adjusting or changing their treatment approach. These results highlighted that the clinical standard manual approach of determining progression mixes objective factors, such as the appearance of new lesions, with other subjective factors such as worsening of neurological symptoms. Thus, there is potential for high variability in RANO judgments between clinicians and between patients even when using the same sources of data. These variable factors could impact results during patient care and data analysis, especially if PFS dates are shared in public data sets without reviewable context on the RANO criteria decision.

### Corticosteroid prescription analysis

Post-radiotherapy steroid prescriptions may provide context about a patient’s disease management that allow clinicians and researchers to further probe for progression evidence. However, in this paper, corticosteroid prescription analysis identified fewer numbers of patients as experiencing progression overall compared to the other manual and data-driven methods. This could be due to patients receiving care management from outside providers after completion of treatment at our center, highlighting barriers that remain within an integrated data framework approach. Conversely, it is important to be cautious when using this method as steroids can be prescribed for non-progression-related reasons, including post-surgical changes. Given that treatment protocols may vary from center to center, it may be appropriate to adjust the date periods in which steroid prescriptions are filtered after surgery and radiotherapy. Thus, steroid-driven progression analysis may include both false positive and false negative errors due to inclusion of non-progression and exclusion of progression-related steroid prescriptions.

This method requires access to patient prescriptions, and can be done with simple data analysis techniques using tabular format data. Moreover, given the finite and structured nature of prescription EHR data, this method required the least amount of data preparation and cleaning. Review of steroid prescriptions also does not necessarily require a specialized clinical expert to query or review the data. As a result, this method may be more straightforward and accessible to non-oncologists researching PFS outcomes in patient cohorts.

However, given that steroids are commonly prescribed for neurological symptoms associated with radiotherapy treatment, it is important to acknowledge that a prescription database may not actually reflect real-world patient medication schedules. It is not uncommon for providers to adjust their dosage and recommendations to patients based on their symptoms after receiving a given prescription. We observed high variability in prescription doses, frequencies, and types of administration in this study (Fig 5c). Given that steroids are also generally prescribed during RT, this prescription data method of determining progression may identify later progression dates if patients hold onto a previous dose of steroids and administer them later on. This disconnect between digital data and real-world behavior remains an issue across multiple areas of clinical research.

### Natural language processing of radiology reports

Ultimately, the rule-based NLP method identified the most number of patients as having progressed in the cohort. While the method displayed the furthest date difference from the clinical standard method, it was also the only method to identify progression in patients with very long stable disease (>100 months) (S2 Fig). These results suggest potential overall benefits from deploying an NLP method, but with a need for further algorithmic design and parameter tuning if close clinical correlation is desired.

The rule-based NLP approach employed in this paper provided a summary of progression-related terms and the context in which they were mentioned for each available report. We opted for a rule-based implementation over other large-scale language models in order to employ a simple, reproducible framework that could be deployed locally. The rule-based approach was also selected to provide improved decision interpretability and reviewability, as the custom progression- and stability-related term matchers allowed researchers to verify progression evidence over the entire course of medical history and seek further context within the original report, if desired (S3b Fig). This method could be embedded into real-world practice where an interested clinician or researcher is provided with an overall graphic interpretation of a patient’s medical history based on these key terms, with the ability to further investigate the actual free text and associated results for time periods of interest. Further research is needed to develop appropriate tutorials for expert users of these systems and evaluate various approaches to report term weighting, evidence presentation, and overall method interpretability in practice.

In order to translate these progression-related terms into a report-level judgment, we weighed terms indicating progression against terms indicating stability or surgical changes. To avoid calling progression too early given the wide range of clinical standard progression patient timelines, we also decided not to weight mentions of progression within report more strongly than mentions of stability or surgical changes. Given high likelihoods of surgical changes being correlated with *pseudoprogression*, we also chose to handle progression-related changes in surgical cavities as indicating “stability” for a patient. The net effect of these choices resulted in a fewer subset of patients in the overall cohort having a report indicating progression.

There was likely some tradeoff in implementing stricter linguistic criteria, as identifying a first date of progression later in long-term stable patients likely came at the expense of identifying progression at all in short-term progressors. It is worth noting that these term formulas could be manipulated in different contexts to give more weight to terms indicating progression versus stability or surgical changes, or a specific subset of terms within each overall category. Adjustments to these formulas may have the net effect of identifying a higher or lower number of progressed patients and/or adjusting the timelines in which patient progression is identified via radiology report. These decisions require judgement as to a preference for high sensitivity or specificity, and the impact of a false positive or false negative may change based on the context that progression data is deployed. Future studies may explore other NLP approaches to mine radiology reports for progression evidence, including the use of large language models (LLMs), algorithms trained with document-level “ground truth” labels for overall progression and stability, and evaluation of the area under the receiver operating curve in order to determine optimal formula weighting and thresholds.

### Computer vision analysis of MRI scans

Imaging reflects a patient’s real-time disease state and can be used in the clinic to guide treatment decisions for a given patient. Radiomic algorithms may provide increased quantitative evidence for decisions in the clinic, as volumetric parameters may be difficult to estimate in practice given the limitations of viewing only two dimensions of a 3D scan slice at any point in time. Human intuitions about volumetric imaging can be subject to errors due to differences in search techniques and cognitive load [82]. The ability to identify regions of contrast-enhancing tumor, non-contrast-enhancing tumor, and edema in a scan closely reflects current clinical imaging practices dictated by RANO progression criteria.

In this paper, we set out to examine the influence of increases in **contrast-enhancing tumor** regions given the independence of growth in relation to steroids and its inclusion within RANO criteria. Perhaps surprisingly, only around half of patients actually progressed by definition of a ≥5% increase in contrast-enhancing tumor despite a majority of patients progressing by manual clinical standards. Our findings align with Kickingereder *et al*., who also observed reduced patient progression rates when comparing increases in contrast-enhancing tumor volumes to manual RANO assessments [19]. This suggests practical differences between the way that RANO criteria are implemented in clinic and how contrast-enhancing tumors manifest on imaging, both volumetrically and perceptually. Given that all but one patients progressed under the manual clinical standard criteria, our scan-based progression findings indicate that clinicians may be overestimating the growth of tumor volumes on scans, or that they are often using other RANO criteria, including worsening clinical symptoms, to determine progression. These results suggest a gap between the underlying logic of RANO criteria and how the clinical principles are applied in practice.

### Comparative analysis

Defining tumor progression is a critical, yet imperfect challenge in cancer management and treatment. The ability to “objectively” determine progression is limited by complex, poorly understood cancer biology and tumor proliferation mechanisms. As a result, any attempt to determine tumor progression within a patient will amount to an imperfect proxy of the underlying ground truth state. Given diverse motivations to study tumor progression, the ideal definition and data points of interest will likely shift between audiences.

Overall, automating progression from only one type of EHR data often resulted in an earlier progression date compared to the manually determined ground truth. If these automated PFS metrics were consulted during clinical treatment, this could result in earlier implementation of more drastic interventions, such as potential re-irradiation or initiation of other therapeutic agents. If the same methods were implemented retroactively during data analysis, earlier progression dates would imply that certain subpopulations of patients had more aggressive disease.

Given that the scan-based progression method identified almost all progressed patients earlier than by clinical criteria, but also identified the fewest number of patients experiencing any progressed, the scan-based method demonstrated a propensity to commit both false positive and negative errors. This indicates that the CV method may benefit from a more nuanced definition of progression, such as taking into account the initial tumor volume size, or incorporating factors of non-contrast-enhancing tumor tissue or edema into future scan-based progression methods.

The report-based method also identified patients as progressing earlier, with the most number of patients being identified compared to the other automated data-driven methods. Given the rule-based nature of the NLP method deployed in this paper, report-level decisions could be adjusted based on the disease aspects most relevant to a given research team. Thus, these results indicate the distribution of outcomes from an automated endpoint extraction framework can be shaped by both data source and algorithmic design.

### Sociotechnical considerations

Human patient and clinician behavior may interact with the design of information systems to shape the process of ground truth construction and extraction of outcome endpoints from EHR data.

#### Changing practices over time.

The use of RANO criteria, radiology reports, and steroid prescriptions all reflect attempts to use human behavior as a proxy for a biological process. Human behavior is cataloged into the electronic health record, either by structured fields via medication prescriptions or by unstructured text via radiology reports and progress notes. As a result, these measures can only capture decisions made in the real world and may undergo “dataset shift” [66] when reflecting medical practices and choices made at the time [27]. Given the relatively small size of the data set in the study, future studies may opt to analyze changes in term, frequency, and prescription patterns over time.

#### Tradeoffs between data modalities.

Many patients in the studied cohort had far more radiology reports available for analysis compared to actual imaging scans. This is potentially surprising given that radiology reports are an interpretation of the processed imaging file and thus, are a degree removed away from the original data source. We speculate that the increased accessibility of radiology reports may be due to patient choices in cancer management. If patients are choosing to continue follow-up care at local facilities, it is possible that current data sharing infrastructure better supports the distribution of radiology reports compared to raw or processed imaging files.

Many available, pre-processed images in the study had to be excluded due to poor image quality or inability of the trained CV algorithm to identify appropriate areas of contrast enhancing and non-contrast enhancing tumor. Moreover, the high number of radiology reports that did not correspond to an available scan indicates existing infrastructure challenges in sharing and querying imaging files. Clinicians and researchers looking to automate PFS via quantitative tumor volume parameters may be limited to fewer datapoints in their analysis given the higher processing burdens of imaging. Conversely, researchers may prefer the use of other higher frequency data types to provide a more continuous picture of a patient’s disease [83].

### Limitations

*Pseudoprogression.* Tumor progression can be difficult to objectively determine for a patient as patients may exhibit signs of pseudoprogression immediately following treatment. When conducting document-level analysis, a patient may demonstrate progression in one scan, a slowing down of progression in a following scan, and then a reversal of slowed progression in the future. This can make it more challenging to rely on a single document to obtain progression data given the importance of context during clinical evaluation. Thus, a framework relying on multiple points of data, such as CV-based volumetric imaging changes, may make it easier to identify between visit changes such as pseudoprogression and stability from a previous progression instance.

*Application of RANO criteria.* One limitation of the study could be the application of RANO criteria and its use as a benchmark against other automated methods. While RANO criteria are the current clinical gold standard, their application requires clinical context that may not have been retrospectively queryable within a system’s EHR. The application of the criteria is a subjective, collaborative process during which we did not have access to individual physician datapoints and thus, were not able to report interrater reliability or agreement on the application of RANO criteria. Bulk analysis methods may omit documents that are not available via a queryable framework, such as scanned, faxed, and/or handwritten notes from historical charts. Radiation treatment plan data was not available at the time of analysis, so 80% isodose lines could not be used to verify progression versus pseudoprogression when evaluating growth in the size or number of lesions. However, given that most radiologists do not have access to this data either, this limitation closely mirrors and reflects real world practice. Future studies may incorporate non-digital documents and radiation treatment plan data to evaluate the extent to which progression versus pseudoprogression is actually captured by observing changes within and outside of the isodose lines.

*Report-level ground truth for NLP.* We were constrained by time- and expert-related resources in obtaining report-level ground truth for the nearly 2,000 radiology reports analyzed in this study. Given that the treatment response and disease progression timeline can vary greatly between patients (e.g., one patient demonstrating no change consistently until a given scan indicates a significant change vs. another patient with alternating periods punctuated by slow change and stability), we were not able to identify a satisfactory proxy in determining the overall evidence for progression or stability in a given report. As a result, we were not able to refine or test our selected one-to-one threshold weighting for terms indicating progression or stability. Future studies could curate (or when possible, employ any newly available public) datasets with report-level ground truth to test and robustly benchmark various rule-based weightings to obtain overall report-level progression determinations.

*Inferring behavior from data.* The analysis of post-radiotherapy steroid prescriptions may have been limited by the fact that we only had access to visits conducted at our medical facility. It is possible that patients may have been received medications, scans, and visits from outside providers. Additionally, given varying practices in tapering prescription schedules for steroids, it was difficult to draw finer insights from differences in prescribed doses or lengths of tapering schedules. This reflects challenges of siloed medical data systems and limits the ability of queryable data frameworks to better approximate “ground truth” determinations.

*Data cohort.* The collection of data at the NIH may also reflect a more unique context in which patients are diagnosed, treated, and managed for complex diseases. Given that patients are often referred from other centers to the NIH where treatment is not associated with insurance billing, it is difficult to assess the representativeness and generalizability of data sets collected at this institution, compared to the general population of individuals affected by a given disease. To our knowledge, this is the first paper that attempts to collect and contrast different modalities of data in order to obtain a subjective patient outcome, and there are no other publicly available data sets to validate this approach yet. Future studies may try incorporating data available outside the NIH, such as radiology reports authored by different clinicians or insurance billing codes, to validate and probe differences in data sets generated between institutions.

*Missing clinical context and accountability.* Some may have valid concerns with non-specialized researchers making progression determinations from the only data that they have available. Further work needs to explore the explainability and interpretability of NLP- and CV-based methods to obtain progression from free-text documents and imaging. There may also be concerns that using single data sources, such as prescriptions or free text documents, may inadvertently result in individuals ignoring relevant information contained in other data modalities. Efforts to improve data sharing and integrated frameworks also need to consider privacy and security concerns when attempting to aggregate large, multi-site sources of data for a given patient.

*Single stream analysis.* Lastly, it is worth noting that all of these automated data-derived progression methods focused on using only one type of data, while the multidisciplinary team clinical standard method incorporated multiple data sources in the EHR to manually determine progression. This paper intentionally set out to focus on the abilities and limitations of individual data sources in identifying “ground truth” within a patient’s clinical history timeline. This decision was made to approximate many real-world clinical scenarios where complete, integrated datasets are not available and difficult to compile and curate. However, with these insights in mind, future studies may investigate multimodal learning techniques to provide progression free survival dates based on a totality of available patient data, including late stage fusion, or aggregation, of the individual models developed for this paper.

## Conclusion

Progression free survival (PFS) is a critical yet under utilized endpoint during biomarker analysis of various malignancies. The current clinical standard to determine progression within a glioblastoma patient involves the application of RANO criteria, a composite of clinical events and imaging findings, during consultation with a multidisciplinary team. This paper set out to explore the benefits and challenges associated with mining different EHR data modalities and automating the extraction of progression free survival metrics via machine learning algorithms. We developed three separate methods to automatically identify progression within a cohort of 92 glioblastoma patients treated on study at the NIH, including 1) selection of categorical corticosteroid prescriptions, 2) rule-based natural language processing of free text radiology reports, and 3) computer vision-based volumetric analysis of brain MRI scans.

Though all three methods were able to provide a progression date for a majority of the patient cohort, they identified fewer patients as having progressed overall compared to the manual clinical standard. Steroid prescriptions were more likely to identify progression later than the manual clinical standard, while CV-based volumetric scan and NLP-based report analysis identified progression much earlier. Approximately half of analyzed patients did not an increase in tumor volumes, indicating that human intuitions about tumor changes during disease progression may not align with quantified volumetric parameters. Our results suggest that various EHR data modalities can be queried to automate PFS analysis, though algorithm design choices, including data modality and progression parameters, will have downstream impacts on clinical decision making or biomedical analysis. Future research directions may explore the benefits and challenges of integrating multiple EHR data modalities, also known as multi-modal analysis, during automated analysis.

## Supporting information

S1 Fig Clinical standard timeline. Regression indicates that there is no statistical relationship between total PFS and time of treatment.
(PDF)

S1 Table Custom NLP pipeline terms. Terms flagged in the progression and stable category, as well as additional modifier terms added to the negation, historical, and custom surgical contextual pipelines. An asterisk (*) indicates that any stem related to or lemma derived from the term was captured.
(PDF)

S2 Fig Progression free survival dates for all data-driven methods by patient. Patients are listed in descending order of clinical standard PFS.
(PDF)

S3 Fig a) Term frequency statistics from local, customized spaCy-based NLP of radiology reports. b) Example term timeline with sentence-level context. (PDF)

S4 Fig Boxplot distributions for volumes extracted from brain MRI scans. All volumes are reported in *cm*^3^. NE tumor = non-contrast-enhancing tumor, CE tumor = constrast-enhancing tumor, Total tumor = NE tumor + CE tumor, Total burden = Total tumor + Edema.
(PDF)

S2 Table Descriptive summary statistics for CV-computed tumor volumes across all available MRI brain scans. All volumes are reported in *cm*^3^. NE tumor = non-contrast-enhancing tumor, CE tumor = constrast-enhancing tumor, Total tumor = NE tumor + CE tumor, Total burden = Total tumor + Edema.
(PDF)
