# Supplementary figures and images for: From manual clinical criteria to machine learning algorithms: Comparing outcome endpoints derived from diverse electronic health record data modalities
Source: PLOS Digit Health. 2025 May 14;4(5):e0000755. doi: 10.1371/journal.pdig.0000755 (PMC12077705; doi:10.1371/journal.pdig.0000755)

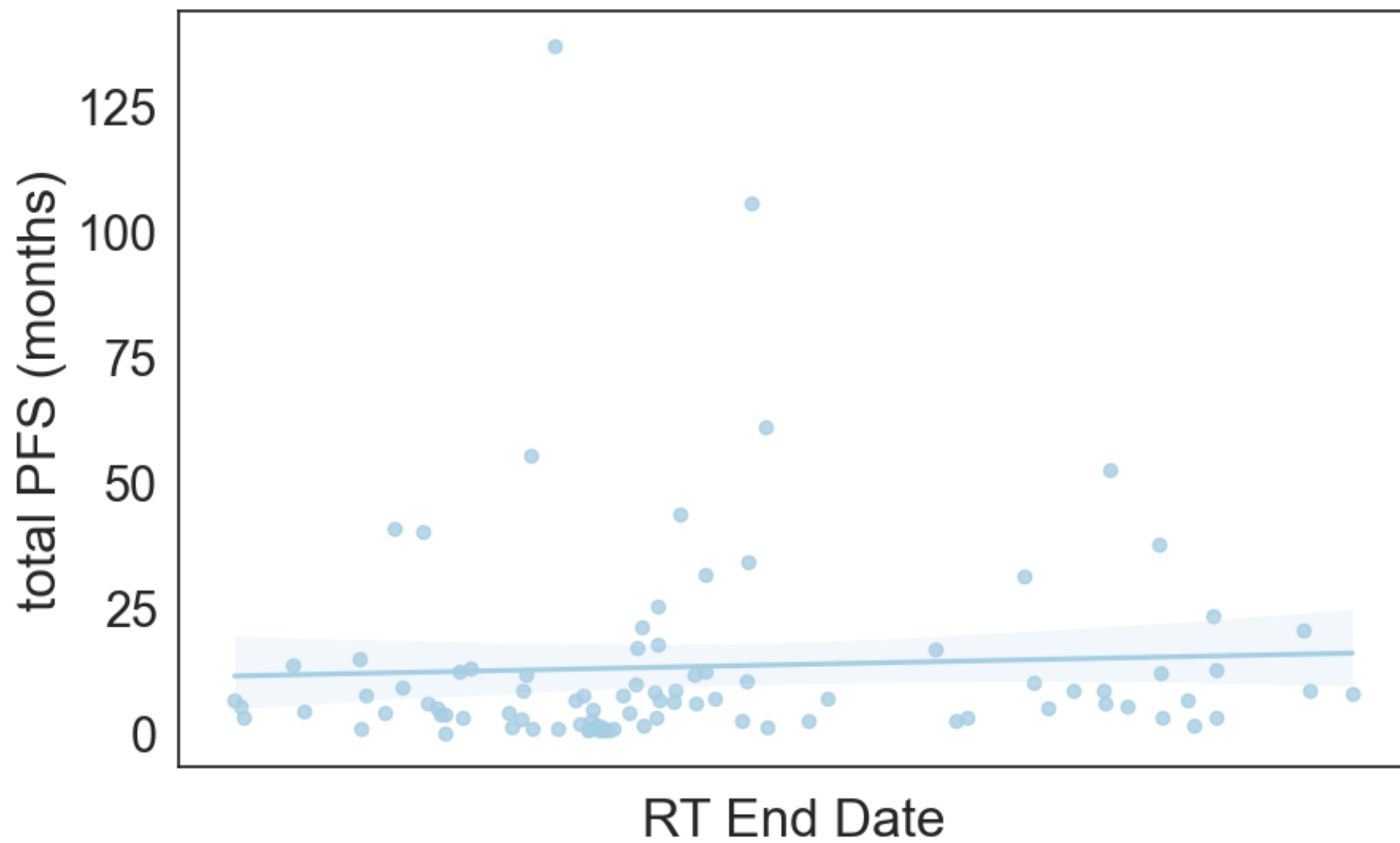

Supplement: S1 Fig — Regression indicates that there is no statistical relationship between total PFS and time of treatment. (PDF) [file pdig.0000755.s001.pdf]

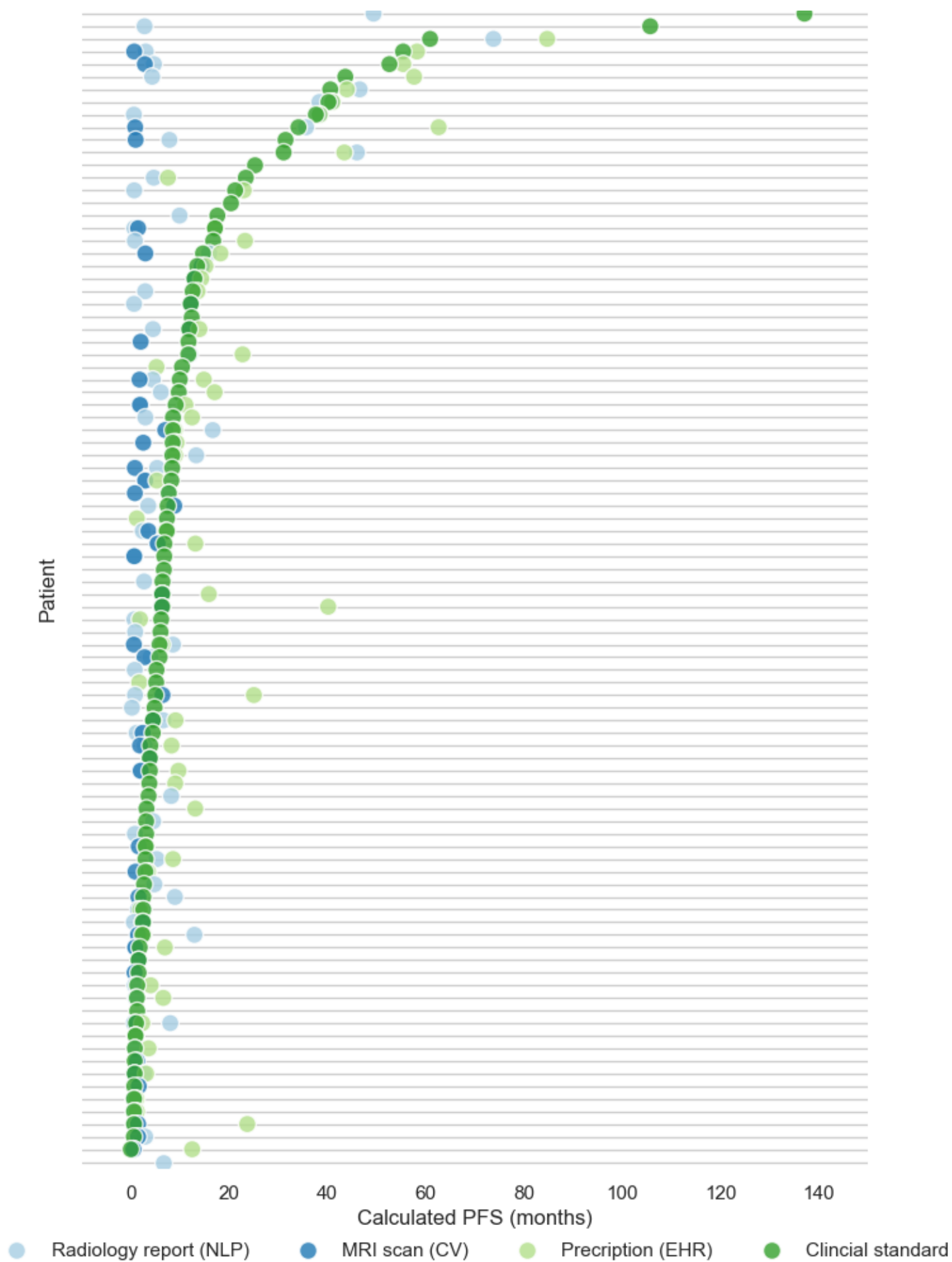

Supplement: S2 Fig — Patients are listed in descending order of clinical standard PFS. (PDF) [file pdig.0000755.s003.pdf]

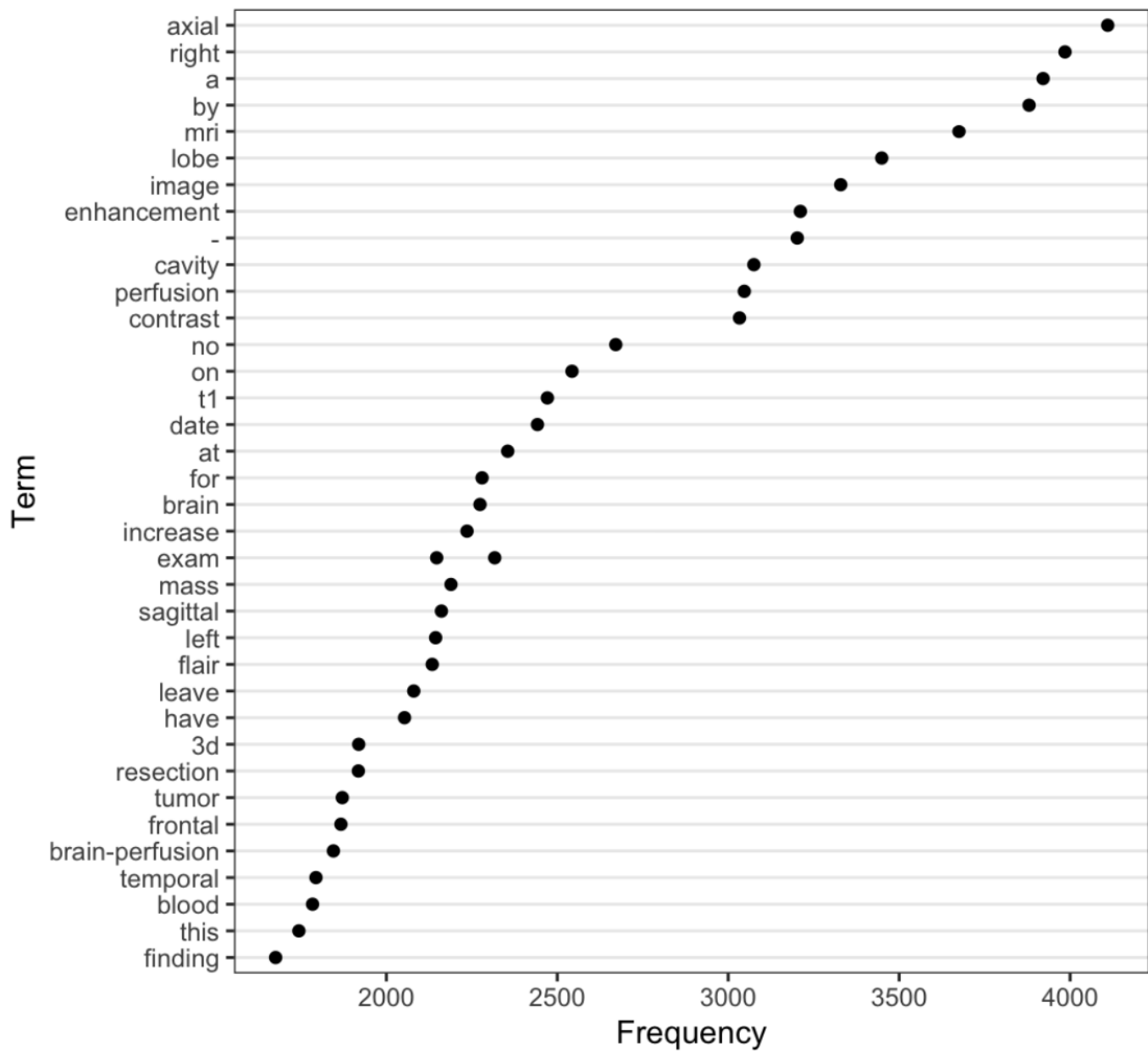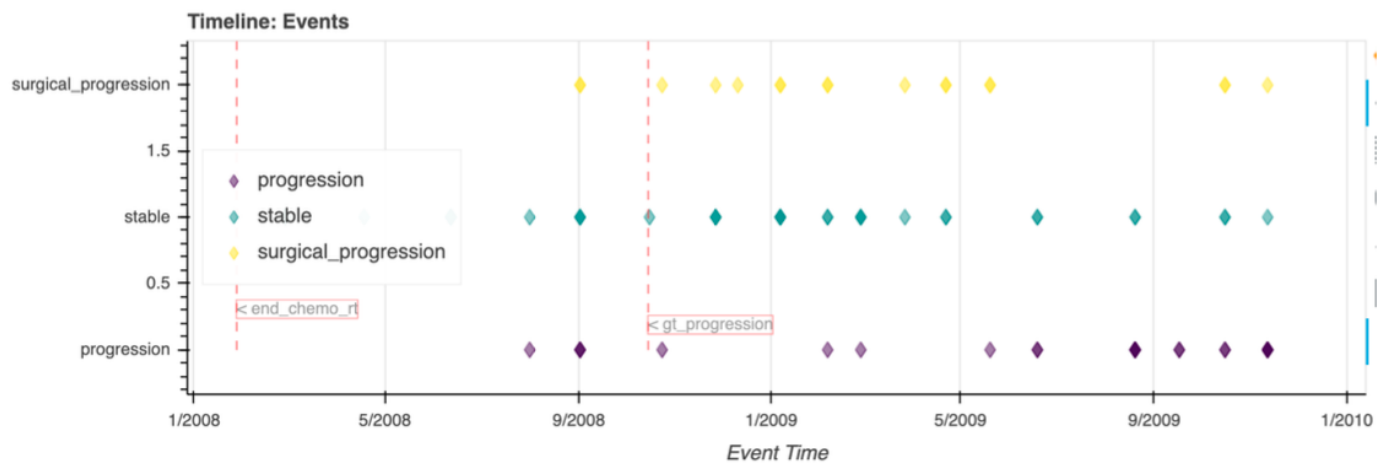

Supplement: S3 Fig — (PDF) [file pdig.0000755.s004.pdf]

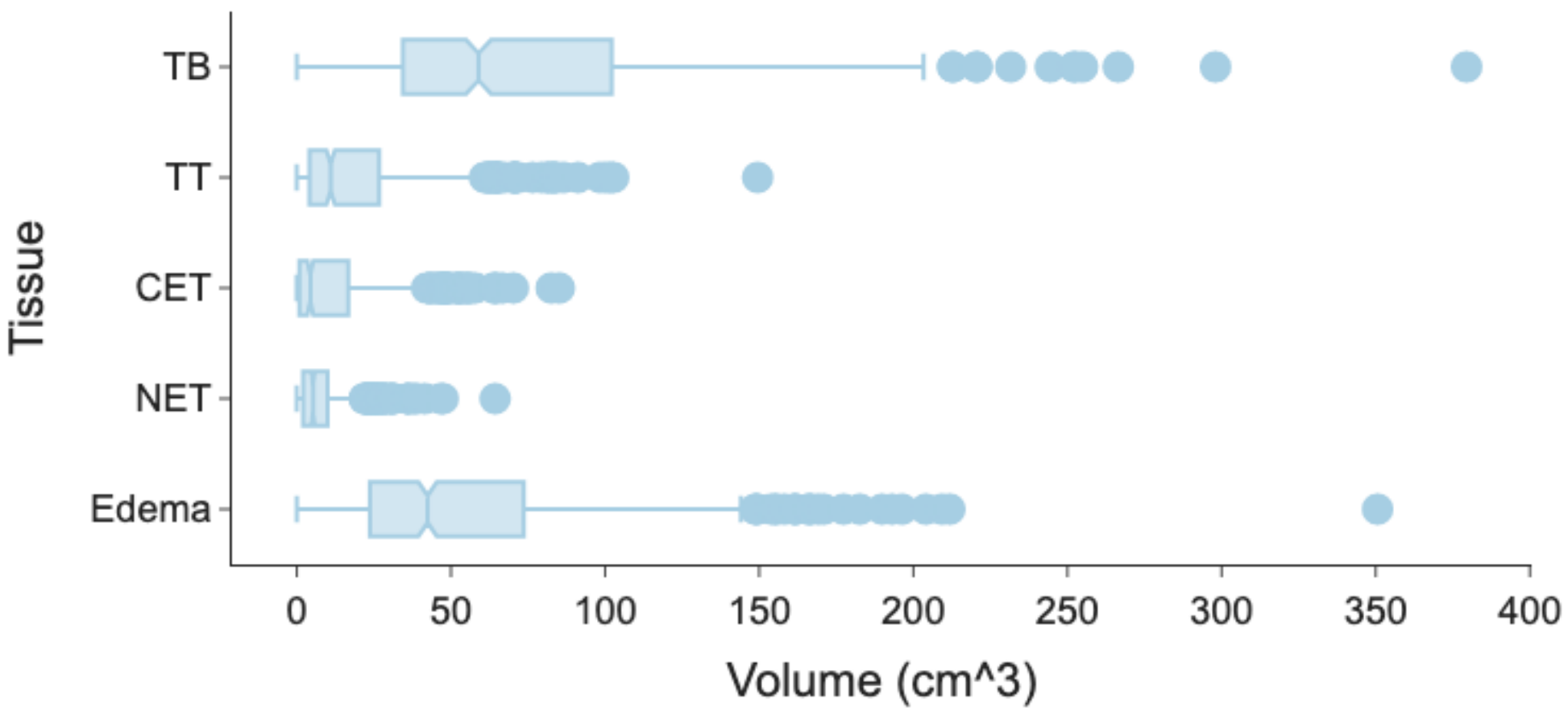

Supplement: S4 Fig — All volumes are reported in cm3. NE tumor = non-contrast-enhancing tumor, CE tumor = constrast-enhancing tumor, Total tumor = NE tumor + CE tumor, Total burden = Total tumor + Edema. (PDF) [file pdig.0000755.s005.pdf]
