# Supplementary material for: From manual clinical criteria to machine learning algorithms: Comparing outcome endpoints derived from diverse electronic health record data modalities
Source: PLOS Digit Health. 2025 May 14;4(5):e0000755. doi: 10.1371/journal.pdig.0000755 (PMC12077705; doi:10.1371/journal.pdig.0000755)
Supplement: S1 Table — Terms flagged in the progression and stable category, as well as additional modifier terms added to the negation, historical, and custom surgical contextual pipelines. An asterisk (*) indicates that any stem related to or lemma derived from the term was captured. (PDF) [file pdig.0000755.s002.pdf]

progression	stable	negated existence	surgical context	historical context
enhancement<br>worsen*<br>progress*<br>enhancement<br>recurrence	unchanged<br>stable<br>further evaluation<br>followup<br>follow up<br>follow-up<br>f/u	no<br>decreased<br>minor<br>minimal*<br>decreas*<br>stable<br>unchang*<br>less<br>small*<br>weak<br>stabiliz*<br>resolv*<br>slight<br>not<br>diminish*	surgical<br>postsurgical<br>postop<br>resection cavity<br>postoperative<br>post op	previous*
