# Supplementary material for: From manual clinical criteria to machine learning algorithms: Comparing outcome endpoints derived from diverse electronic health record data modalities
Source: PLOS Digit Health. 2025 May 14;4(5):e0000755. doi: 10.1371/journal.pdig.0000755 (PMC12077705; doi:10.1371/journal.pdig.0000755)
Supplement: S2 Table — All volumes are reported in cm3. NE tumor = non-contrast-enhancing tumor, CE tumor = constrast-enhancing tumor, Total tumor = NE tumor + CE tumor, Total burden = Total tumor + Edema. (PDF) [file pdig.0000755.s006.pdf]

	**Edema**	**NE tumor**	**CE tumor**	**Total tumor**	**Total burden**
**mean**	53.5	7.5	11.0	18.5	72.0
**std**	41.6	7.8	14.5	20.2	52.4
**median**	42.4	5.2	4.4	11.0	59.0
**range**	0-350.6	0-64.3	0-85.2	0-149.5	0-379.5
